# Supplementary material for: Staphylococcus aureus Protection-Related Type 3 Cell-Mediated Immune Response Elicited by Recombinant Proteins and GM-CSF DNA Vaccine
Source: Vaccines (Basel). 2021 Aug 13;9(8):899. doi: 10.3390/vaccines9080899 (PMC8402413; doi:10.3390/vaccines9080899)
Supplement: Supplementary file 1 [file vaccines-09-00899-s001.zip › vaccines-1259985-supplementary.pdf]

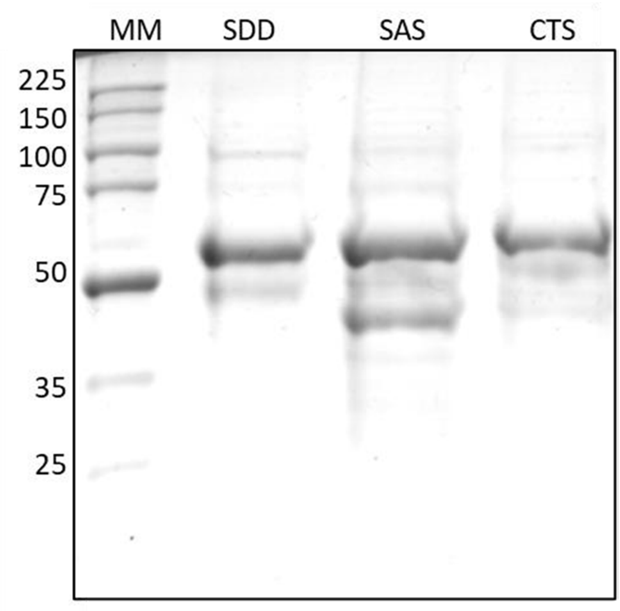

Supplementary Figure S1. SDS-PAGE analysis of *Staphylococcus aureus* recombinant proteins. Coomassie blue-stained 12% SDS-PAGE analysis of cell lysate insoluble fraction and purified fractions resulting from IPTG-induced bacterial cultures showing bands in the expected sizes for S. aureus recombinant proteins. MM: molecular mass in kDa. Bands of approximately 52.5 kDa, 56.2 kDa and 52.7 kDa referring to succinyl-diaminopimelate (SDD), F0F1 ATP synthase subunit α (SAS) and cysteinyl-tRNA synthetase (CTS) proteins were observed, respectively. SDS-PAGE: Polyacrylamide Gel Electrophoresis; IPTG: Isopropyl β-d-1-thiogalactopyranoside.

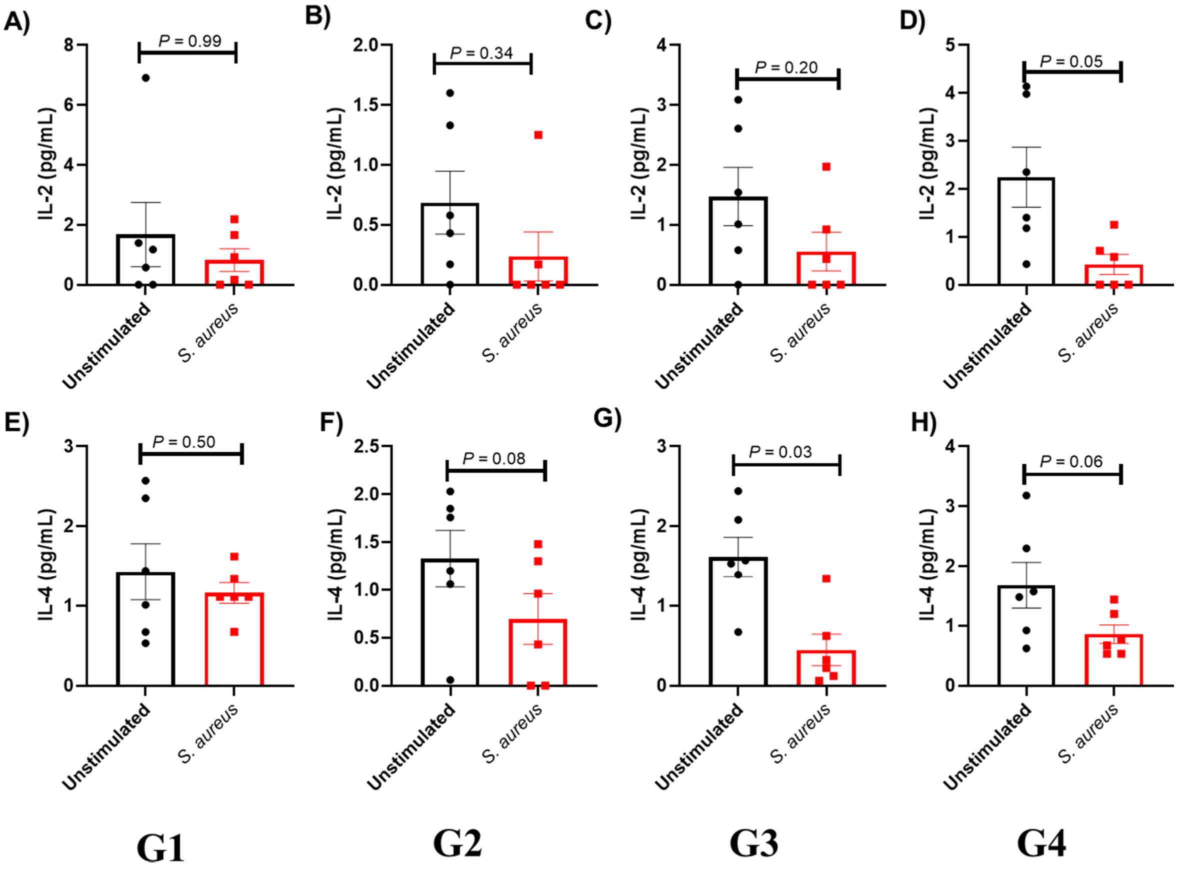

Supplementary Figure S2. Cytokine concentration in the supernatant of the immunized and non-immunized mouse spleen cells culture under unstimulated (basal) and *Staphylococcus aureus* stimulated conditions. IL-2: interleukin-2; IL-4: interleukin-4; G1: unvaccinated control group; G2: GM-CSF DNA plasmid DNA vaccination; G3: SAS+SDD+CTS *S. aureus* recombinant proteins vaccination; G4: SAS+SDD+CTS *S. aureus* recombinant proteins vaccination associated with GM-CSF DNA plasmid DNA vaccine; SAS: F0F1 ATP synthase subunit α; SDD: succinyl-diaminopimelate; CTS: cysteinyl-tRNA synthetase. [Student t test (A, D, E, F, G and H) and Wilcoxon test (B and C)].

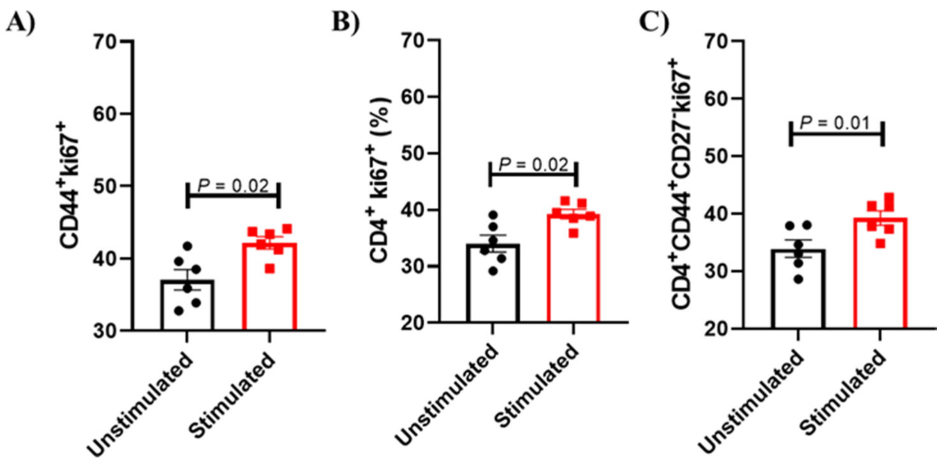

Supplementary Figure S3. Proliferative responses of lymphocytes in mice vaccinatedwith the three recombinant proteins and the GM-CSF DNA vaccine. **A)** Percentage of proliferative (ki67$^+$) overall memory T cells (CD44$^+$), **B)** CD4$^+$ cells, and **C)** CD4$^+$ T effector memory (CD44$^+$ CD27$^-$) under unstimulated and *S. aureus* stimulated conditionsin mice (n = 6) vaccinated with the three recombinant proteins and the GM-CSF DNA vaccine. GM-CSF: Granulocyte-Macrophage Colony-Stimulating Factor. [Student t test].
